# Contraception and PrEP knowledge, attitudes, and practices among adolescent girls and young women in Nampula, Mozambique

**DOI:** 10.1371/journal.pgph.0004746

**Published:** 2025-06-16

**Authors:** Allison Hsu, Joana Falcao, Ricardino Zandamela, Allison Zerbe, Jennifer M. Zech, Eduarda Pimentel de Gusmao, Elizabeth Stephanz, Mirriah Vitale, Elaine J. Abrams

**Affiliations:** 1 ICAP at Columbia University, New York, New York, United States of America; 2 ICAP at Columbia University, Maputo, Mozambique; 3 Vagelos College of Physicians and Surgeons, Columbia University, New York, New York, United States of America; University of Toronto, CANADA

## Abstract

Despite widespread availability of ART and PrEP, HIV incidence among adolescent girls and young women (AGYW) in Mozambique remains amongst the highest in Africa. Similarly, modern contraception methods are broadly available in the public sector, but high rates of unplanned pregnancies persist. We explored AGYW’s sexual behavior and knowledge of sexual and reproductive health, and HIV prevention practices in Nampula, Mozambique. Between May-June 2022, we conducted a cross-sectional survey among a convenience sample of AGYW 15–25 years whose self-reported HIV status was negative or unknown receiving care at three adolescent health clinics in Nampula province. Information on demographics, sexual behavior, HIV, and contraception and PrEP knowledge and attitudes was collected. Responses were analyzed using descriptive statistics. Of 200 AGYW (median age: 19 years, interquartile range: 17-21.3 years), 55% were in school, 32% completed secondary school and 81% had never been employed. Overall, 77% reported ever having had sex and 26% reported being <16 years at first sex. Nearly all respondents knew about condoms; only half had used a condom at last sex. Male condoms (62%), withdrawal (35%), and implants (25%) were the most commonly previously used contraceptive methods. AGYW were most interested in trying implants (18%), injectables (12%), and the pill (11%), though 31% reported not being interested in trying any new contraceptive method. Respondents had accurate general HIV knowledge; only 26% reported had ever heard of PrEP and 2% reported previous use. However, 64% expressed interest in using PrEP daily pills and 76% in long-acting injectables. AGYW in Nampula reported low usage of modern contraceptive methods and poor knowledge of PrEP. AGYW expressed interest in trying contraception and PrEP and showed positive attitudes toward PrEP usage. More widespread education around family planning and HIV prevention is needed to reduce barriers around improving sexual health among AGYW in Mozambique.

## Introduction

Despite great advances across the HIV care continuum, with increased numbers of people living with HIV on antiretroviral therapy, efforts to prevent new infections have been less successful than expected. In particular, HIV prevention efforts among adolescents and young adults (AYA) in sub-Saharan Africa (SSA) have been met with only modest success, with approximately 63% of all new HIV infections in Eastern and Southern Africa occurring among young people [1]. In particular, young women aged 15–24 years represent 10% of the SSA population but 25% of new HIV infections [2].

Mozambique is a country in SSA with high HIV prevalence (12.5%) and incidence, with 98,000 new infections in 2021 [2,3]. Of these, 56% were among women [2]. The annual HIV incidence among AYA was 0.50, the highest among all age groups, and the incidence among AGYW was 0.72 [3]. The province of Nampula, with the largest general population and the largest AYA population, accounted for 20% of all new HIV infections [4]. AGYW in Mozambique remain at high risk for HIV acquisition due to gender norms that make insisting on condom use difficult [5] as well as barriers to access – 26% of young women aged 15–24 years report an unmet need for family planning services [6]. High rates of physical violence [7] and low rates of school attendance [8] further put young women at risk for HIV acquisition.

Extensive data support the association between early sexual debut and increased HIV risk [9]. In Mozambique, sexual debut is early among AGYW, on average before the age of 16, with 40% of young women becoming pregnant prior to the age of 18 [10,11]. Risky sexual behaviors such as sex at an early age can also increase the risk of unplanned pregnancy [12]. In 2023, the rate of unplanned pregnancies among adolescents aged 15–19 in Mozambique was 180 per 1000 girls [13]. Across East African countries, uptake of modern contraceptives is estimated to be about 27% with high rates of discontinuation [14]. In Mozambique specifically, contraceptives are broadly available in the public sector but roughly 50% of women who initiate contraception discontinue within 12 months [14]. Reasons for discontinuation include method failure, desire to get pregnant, and side effects of the contraceptive method [14]. Additionally, only about half of young women report using a condom with a non-regular partner [15].

In Mozambique, the national roll-out of daily oral antiretroviral pre-exposure prophylaxis (PrEP) began in October 2021. AYA, and AGYW in particular, were identified as a priority population by the Ministry of Health, given high HIV incidence and modeling data that project substantial impact of PrEP on averting new infections among AGYW [8,16,17]. However, programmatic data for the first year of the national rollout show that while almost 56,000 clients initiated PrEP between October 2021 and June 2022, only 20% were found to have continued PrEP (as measured by a record of any follow-up visit) [18]. This suboptimal retention highlights the need to better understand individual, interpersonal, and health system/institutional barriers that impact PrEP uptake and continuation and the importance of tailoring PrEP services to the unique needs of specific target populations, including AGYW [19]. Additionally, a nationwide survey that was done around the same time as the national PrEP roll-out reported that only 6.6% of AGYW aged 15–24 years had heard of PrEP [3], much lower than other countries in the region [20–22]. Looking forward, lessons learned may then be applied to increase awareness, uptake, and continuation as the available PrEP choices expand in Mozambique to include long-acting injectables and the vaginal ring [23].

The aim of this study was to understand the specific attitudes, knowledge, and practices of AGYW in Nampula, Mozambique, around sexual and reproductive health as well as identify the barriers that prevent these AGYW from accessing or using sexual and reproductive health services. While some data exists that speak to contraceptive and PrEP use among AGYW in this area, there are gaps in our understanding. Assessing and understanding current behaviors and preferences of AGYW in high HIV burden areas of Mozambique may aid in the development of new interventions or the refinement of existing programs to improve uptake of sexual and reproductive health services with a particular focus on PrEP uptake and adherence, such as integrating PrEP into established family planning venues. Additionally, understanding the differences between younger AGYW (15–19 years) and older AGYW (20–25 years) will inform specialized programming to further improve service uptake.

## Methods

### Participants and procedures

We conducted a cross-sectional survey among AGYW aged 15–25 years receiving care in the Adolescent and Youth Friendly Service clinics (SAAJ), in three urban government health facilities in Nampula, Mozambique, recruiting participants between 19/05/2022 and 14/06/2022. Within government health facilities, SAAJ clinics are staffed with healthcare workers specifically trained in adolescent HIV prevention/care and sexual and reproductive health, providing services on a flexible schedule in a dedicated space. These clinics aim to be a one-stop-shop for adolescents providing free counseling on sexual and reproductive health, antenatal and postnatal visits for pregnant clients, prevention services, as well as substance use prevention, gender-based violence services, and HIV and STI testing and treatment services [24]. All AGYW aged 15–25 years who were receiving care at the SAAJ clinic during the recruitment window and self-reported as HIV-negative or unknown HIV status were eligible to participate and were approached for participation in the survey. During this window, SAAJ staff identified potential participants present in the SAAJ waiting area, introduced the study, and referred interested AGYW to trained study staff based at the health facility. Recruitment was complete once the sample size was achieved.

Study staff reviewed eligibility criteria (aged 15–25 years, reported HIV-negative or unknown status) and obtained written informed consent from participants >18 years of age. For AGYW under 18 years old, study staff obtained written informed consent from their adult caregivers as well as written informed assent from the participant. For all participants, informed consent and assent procedures as well as survey administration were conducted in Portuguese within private spaces in SAAJ facilities.

All study staff were female aged 18–25 years and were experienced research interviewers. Study staff administered the tablet-based survey by reading the questions and response options to the participants and selecting the participants’ responses directly on the tablet. To educate participants about the different contraception and PrEP modalities, descriptions included in the survey tool were read aloud by study staff. The contraception modalities explained were injectables, implants, the pill, intrauterine device (IUD), female condom, emergency contraception, standard days method, lactational amenorrhea method (LAM), and withdrawal. Described PrEP modalities included daily oral PrEP, on-demand oral PrEP, long-acting injectable PrEP (IM-LA), and the PrEP vaginal ring.

Participants received the equivalent of about 5 USD in Mozambique currency (300 Meticais). For participants aged 15–17 years, caregivers received an additional 4 USD in Mozambique currency (200 Meticais) for their time and effort.

### Study measures

The survey consisted of 134 close-ended, multiple-choice questions and 10 open-ended questions. Survey domains included demographics, education, employment, household characteristics, relationships and pregnancy, sexual behavior, contraception knowledge, HIV testing experience, HIV knowledge, PrEP knowledge and attitudes, PrEP stigma, and PrEP modality preference and service delivery.

Demographic questions were derived from both The Demographic and Health Surveys (DHS) Program’s woman’s questionnaire and the CombinADO survey, which had been previously validated in Mozambique [25,26]. The DHS survey was also used to inform questions related to personal relationships and pregnancy [26]. The sexual behavior section was informed by illustrative core instruments specifically developed by Cleland and colleagues to ask young people about sexual and reproductive behaviors [27]. The Global School-Based Student Health survey (GSHS) was also used to develop questions in this section [28]. The DHS survey was also used to derive questions about contraception knowledge and HIV testing experiences [26]. The GSHS was used to derive fourteen ‘true’/’false’/’don’t know’ questions to assess knowledge about HIV and HIV transmission [28]. Questions regarding PrEP knowledge, attitudes, and practices were derived using a tool developed by Sila et al, which was field-tested prior to research use [29]. The Sila survey was also used to inform the PrEP stigma section [29]. We evaluated PrEP stigma with six statements around PrEP use and asked the participants to rate their agreement with the statement using a five-point Likert scale [29]. Questions from the DHS survey were also used to develop the questions regarding PrEP modality by using the contraception questions and applying the same concept to PrEP [26]. The survey sources are summarized in S1 Table.

### Data analysis

We present descriptive data on the characteristics of AGYW 15–25 years of age. Data analysis was conducted using SAS OnDemand for Academics (SAS Institute Inc., Cary, NC, USA). The initial analysis consisted of determining the counts and frequencies for each survey question. We also examined characteristics according to age group – for this analysis, the younger age group was defined as 15–19 years, and the older age group was defined as 20–25 years, as outlined in the PEPFAR Monitoring, Evaluation, and Reporting Indicator Reference Guide, FY25 [30]. Descriptive and inferential statistics were employed to assess survey responses. Tests of significance for descriptive and inferential statistics (p < 0.05) by age group were conducted using Chi-square or Fisher’s Exact tests for categorical variables. Relative risk ratios (RR), calculated using 2x2 tables, are presented with 95% confidence intervals (CI). A multivariable logistic regression was modeled for modern contraception use (male/female condoms, implants, pill, injectables, IUD, emergency contraception) and participant awareness of PrEP using the following variables: age group, education level, marital status (married, living with partner vs. single, separated, or divorced), and reported number of sexual partners in the past year (0, 1, 2+). Odds ratios (OR) and adjusted odds ratios (aOR) with 95% CIs are reported.

### Ethical approvals

The protocol was approved by the Columbia University Irving Medical Center Institutional Review Board (IRB-AAAT9339) and the Comité Nacional de Bioética em Saúde of the Ministry of Health (233/CNBS/22) in Mozambique.

### Inclusivity in global research

Additional information regarding the ethical, cultural, and scientific considerations specific to inclusivity in global research is included in the Supporting Information (S1 Checklist).

## Results

### Participant characteristics

A total of 200 AGYW completed the survey and are included in this analysis. The median age was 19 years (interquartile range [IQR] 17-21.5 years) (Table 1). Younger AGYW (15–19 years) represented 59.0% of the study sample (median age = 18 years) and 41.0% were older AGYW (20–25 years, median age = 22 years). Over half (57.5%) identified as Catholic or Christian and 26.0% identified as Muslim. The majority (74.5%) reported being single. More than half (54.5%) were currently enrolled in school and 32.0% had completed secondary school. Overall, 80.5% had never worked and only 8.5% were currently working. While 88.0% reported having electricity in their house, only 4.5% reported running water inside the house and 6.0% had inside toilets. The most common source of drinking water was running water in the garden (38.0%). Almost two-thirds (63.0%) of participants had their own cellphone while 16.5% had no regular access to a cellphone. Older participants aged 20–25 years were 2.7 (95% CI: 1.7-4.5) times more likely to have a cellphone as compared to younger participants aged 15–18 years (p-value <0.001).

**Table 1:** Demographic characteristics of adolescent girls and young women (15-25 years) completing the survey in Nampula, Mozambique, May-June 2022 (N = 200).

	All		15-19 years		20-25 years		p-value
	N	%	N	%	N	%	
Age	200		118		82		
Religion							
Christian/Catholic	115	57.5	66	55.9	49	59.8	0.770
Muslim	52	26.0	16	13.6	11	13.4	
Another religion	33	16.5	21	17.3	12	14.4	
Marital status							
Married and living together	23	11.5	11	9.3	12	14.6	0.002
Married and not living together	4	2.0	3	2.5	1	1.2	
Living together but nor married	19	9.5	6	5.1	13	15.9	
Widowed	0	0.0	0	0.0	0	0.0	
Divorced/separated	5	2.5	0	0.0	5	6.1	
Single	149	74.5	98	83.1	51	62.2	
Currently enrolled in school? (N = 197)							
Yes	109	54.5	78	66.1	31	37.8	<0.001
No	88	44.0	38	32.2	50	61.0	
No answer	3	1.5	2	1.7	1	1.2	
Highest level of education (N = 197)							
None	3	1.5	2	1.7	1	1.2	<0.001
Incomplete primary	19	9.6	15	12.9	4	4.9	
Primary/incomplete secondary	94	47.7	76	65.5	18	22.2	
Secondary education	64	32.5	23	19.8	41	50.6	
Technical/vocational education	15	7.5	2	1.7	13	16.0	
University	5	2.5	0	0.0	5	62	
Employment status							
Currently working	17	8.5	9	7.6	8	9.8	0.025
Not working now but worked during the last year	17	8.5	8	6.8	9	11.0	
Not working now but worked prior to last year	5	2.5	0	0.0	5	6.1	
Never worked	161	80.5	101	85.6	60	73.2	
Own cell phone							
Yes	126	63.0	59	50.0	67	81.7	<0.001
No	74	37.0	59	50.0	15	18.3	
Household characteristics							
Non-flushing toilet	53	26.5	27	22.9	26	31.7	
Latrine	128	64.0	83	70.3	45	54.9	
Electricity	176	88.0	102	86.4	74	90.2	
Running water inside	52	26.0	24	20.3	28	34.1	

### Sexual behavior

Overall, 77.0% (N = 154) of participants reported ever having had sex, whether vaginal, anal, or oral sex, or any combination of the three (Table 2). Younger AGYW (15–19-year-olds) were 14.9 (95% CI: 3.7-60.0) times less likely to report a history of sex (p < 0.001). The median age at first sex was 17 years (IQR 15–18), and 26.0% of participants reported being 15 years old or younger at time of first sex. Of those reporting history of sexual activity, 17.5% said that the first time they had sex was forced sex, with 66.7% of those reporting being pressured through harassment, threats, or tricks, and the other one-third being physically forced. There was no significant difference between age groups in report of whether first sex was desired or forced.

**Table 2:** Sexual history and behavior of AGYW (N = 200).

	All (N = 200)		15-19 years (N = 118)		20-25 years (N = 82)		p-value
	N	%	N	%	N	%	
History of sex							
Yes	154	77.0	74	62.7	80	97.6	<0.001
No	45	22.5	43	36.4	2	2.4	
Prefer not to answer	1	0.5	1	0.8	0	0.0	
If reported history of sex, type of sex had (choose all that apply)							
Vaginal	154	100.0	74	100.0	80	100.0	<0.001
Anal	4	2.6	0	0.0	4	4.9	0.015
Oral	34	22.1	9	7.6	25	30.5	<0.001
Median (IQR) age at first sex	median	IQR	median	IQR	median	IQR	
	17	15, 18	16	15, 17	18	16, 19	
First sex willingness							
Wanted to	124	80.5	58	78.4	66	82.5	0.284
Forced	27	17.5	13	17.6	14	17.5	
Don’t know	3	1.9	3	4.1	0	0.0	
How first sex was forced							
Physically forced	9	33.3	5	38.5	4	28.6	0.695
Pressured	18	66.7	8	61.5	10	71.4	
Reason for first sex (choose all that apply)							
I wanted to try it	104	81.9	46	75.4	58	87.9	0.068
My friends pressured me to have sex	17	13.4	8	13.1	9	13.6	0.931
To show my love/to feel loved	82	64.6	34	55.7	48	72.7	0.046
My partner wanted to have sex	97	76.4	44	72.1	53	80.3	0.279
For money or gifts	7	5.5	3	4.9	4	6.1	1.000
I wanted to have a baby	12	9.4	4	6.6	8	12.1	0.284
Other	3	2.4	3	4.9	0	0.0	0.108
I don’t know	2	1.6	2	3.3	0	0.0	0.229
Total number of partners in lifetime							
One	48	31.2	32	45.1	14	18.2	<0.001
Two	45	29.2	23	32.4	19	24.7	
Three or more	61	39.6	16	22.5	44	57.1	
Number of partners in past year							
Zero	6	3.9	4	5.4	2	2.5	.432
One	117	76.0	53	71.6	64	80.0	
Two or more	31	20.1	17	23.0	14	17.5	
Heard of condoms?							
Yes	150	97.4	70	94.6	80	100.0	0.051
No	4	2.6	4	5.4	0	0.0	
Condom used at last sex?							
Yes	75	50.0	39	55.7	36	45.0	0.190
No	75	50.0	31	44.3	44	55.0	
Reason for not using condom during last sex act (choose all that apply)							
I did not want to use condoms	39	52.0	16	51.6	23	52.3	0.955
My partner did not want to use condoms	18	24.0	10	32.3	8	18.2	0.160
I wanted to become pregnant	8	10.7	1	3.2	7	15.9	0.130
I/we did not have a condom	17	22.7	12	38.7	5	11.4	0.005
I/we were drunk	0	0.0	0	0.0	0	0.0	N/A
I/we do not know how to use a condom	2	2.7	1	3.2	1	2.3	1.000
Other	34	45.3	12	38.7	22	50.0	0.333
I don’t know	1	1.3	0	0.0	1	2.3	1.000
Condom use frequency							
Always	33	22.0	20	28.6	13	16.3	0.223
Sometimes	81	54.0	35	50.0	46	57.5	
Never	35	23.3	15	21.4	20	25.0	
I don’t know	1	0.7	0	0.0	1	1.3	
Easy to get condom if wanted?							
Yes	117	76.0	50	67.6	67	83.8	0.048
No	31	20.1	21	28.4	10	12.5	
Don’t know	6	3.9	3	4.1	3	3.8	

Among those AGYW who were sexually active, 39.6% reported having three or more lifetime sex partners, and 25.5% had more than one sex partner in the previous year. The majority of younger AGYW (66.7%) last had sex with someone older than 19 years, while 35.5% of older respondents last had sex with someone older than 25 years. All but four respondents, all in the younger age group, had ever heard of condoms. Half (50.0%) of respondents had used a condom at last sex. The main reasons for not using a condom included one or both partners not wanting to use one (81.3%), not having a condom (22.7%), wanting to become or already being pregnant (17.3%), and having a committed or trusted sex partner (9.3%). Despite 76.0% of participants reporting that it would be easy for them to get a condom if they wanted, only 22.0% of respondents reported always using a condom during sex. Over half of participants (54.0%) said they only sometimes use a condom, and almost one-quarter (23.3%) said they never use a condom. Older AGYW were 1.2 (95% CI: 1.0-1.5) times more likely to report that it would be easy to get a condom if they wanted (p = 0.013).

### Contraception use and pregnancy

Outside of male condoms, most participants were aware of other common contraceptive methods such as implants (95.5%), pills (92.0%), and injectables (85.0%). After male condoms (62.0%), withdrawal was the most common contraceptive method that participants had previously used (35.0%), and 25.0% had experience using implants. Overall, 15 AGYW (7.5%) reported no history of contraception use, though 13 of these participants (87.0%) had reported a history of sex.

About half of participants (48.0%) were currently doing something or using a method to delay or avoid getting pregnant. Male condoms (65.6%), implants (29.2%), and injectables (11.5%) were the three most popular methods reported by these participants. The most common reasons cited for not using any contraceptive method to prevent pregnancy were not having regular sex or having not had sex yet (23.3%), participant or partner being afraid of contraceptives (18.6%), and participant desire to become pregnant (17.4%). Other reasons listed were that the participant was already pregnant (15.1%), either the partner or caregiver refuses contraception (14.0%), and being unfamiliar with where or how to buy contraception (4.7%). In the logistic regression model, participants who were single, divorced, or separated were more likely to use a modern contraceptive method than participants who were married or living with their partner (p < 0.001; OR=4.5; aOR=4.6; 95% CI: 2.0-10.5). All other variables (age, education, reported number of sexual partners in the past year) were non-significant.

When asked which contraceptive methods they would want to try, while 30.5% of respondents said none, almost half of respondents expressed interest in short-acting methods (47.0%), including and injectables (12.0%) and pills (11.0%). Only 19.5% of respondents said they wanted to try long-acting methods; notably only 1.0% expressed interest in IUDs, and the other 18.5% in implants. Many (38.0%) stated that they were afraid of using an IUD. Some AGYW also reported fears associated with using implants (32.5%) and injectables (26.5%). Common reasons for fearing these methods included concerns about implants getting lost in the body (12.0%), belief that contraception (especially IUDs and implants) causes infertility (11.5%), fear of needles (5.5%), and fear of side effects (6.0%). Participants agreed that the most appropriate method of contraception for young people (aged 15–25 years) was male condoms (56.0%), implants (51.0%), and injectables (17.0%).

**Fig 1:**
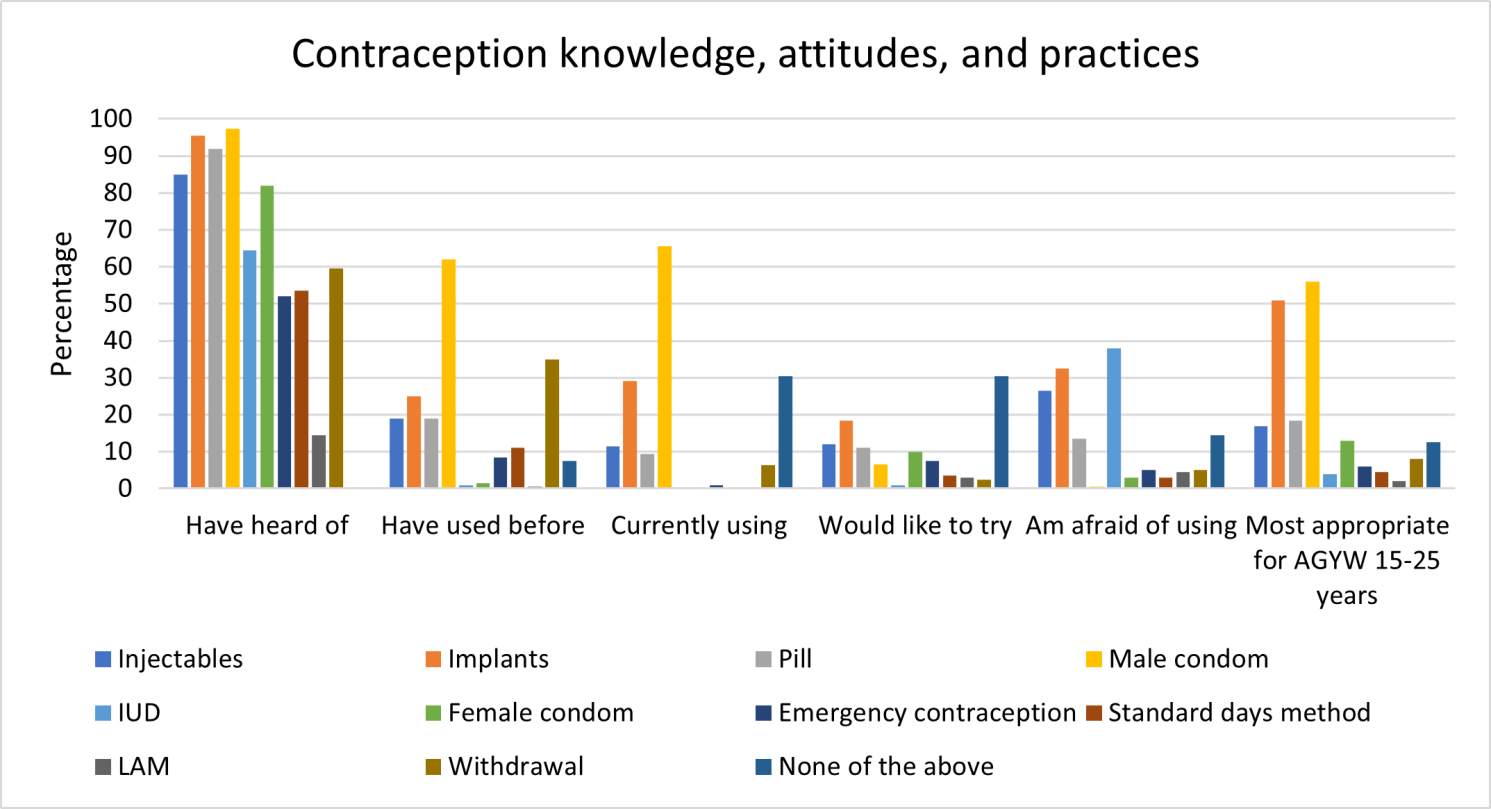
Knowledge, attitudes, and practice of AGYW across different modalities of contraception (N = 200).

Older AGYW were more likely to have heard of and tried each contraceptive method listed in Fig 1. Older AGYW were also significantly more likely to want to try pills (p = 0.022, RR = 2.5, 95% CI: 1.1-5.3). The older age group was 1.8 (95% CI: 1.3-2.5) times more afraid of trying the IUD than the younger age group (p = 0.001), but the differences in fear of trying other contraceptive methods between age groups were not significant. The younger group was much more likely to think that no contraceptive methods are appropriate for AGYW to use (p < 0.001, RR = 8.0, 95% CI: 1.9-33.0). Older AGYW were more likely to report having a family member (p = 0.016, RR = 1.39, 95% CI: 1.1-1.8) or partner (p = 0.007, RR = 2.3, 95% CI: 1.2, 4.2) they could trust to answer questions about sex, pregnancy, and contraceptive methods.

About one-third of all participants had a history of pregnancy (34.1%), with 24 participants (12.0%) being pregnant at the time of the survey. The median age at first pregnancy was 18 years (IQR 17–19 years, range 13–23 years). Of those who had been pregnant (N = 60), about half wanted to become pregnant (45.0%) while the other half did not (55.0%). Also, 35.0% of participants who had a history of pregnancy also reported having had a voluntary abortion. Most (71.4%) of these participants reported receiving abortion services at a health center, but 28.6% reported undergoing their abortion at home, despite the fact that public health services provide abortions free of cost. However, even though 61.7% (N = 37) of those who had history of pregnancy knew that abortion is legal in Mozambique, only six of these participants (16.2%) knew that they were free.

Of those who reported a previous pregnancy (N = 60), only one had never been tested for HIV citing fear as the reason for avoiding the test. Of those who reported a previous pregnancy and had tested HIV-negative, just over half (52.7%) reported being offered counseling or referral to HIV prevention services.

### HIV and PrEP knowledge, attitudes, and practices

The participants were asked fourteen true or false questions about HIV knowledge. Among the group, there was an average score of 10/14 correct (72.0%). The question that most participants answered incorrectly was “People with HIV should hide from others that they have HIV” with only 40.0% answering “false.” Other questions with a low correct percentage were “There is a cure for HIV” (57.0%), “Showering after sex greatly reduces the transmission of HIV” (47.0%), and “A person can get HIV from anal sex” (47.0%). There was not a significant difference in HIV knowledge between younger and older age groups.

**Fig 2:**
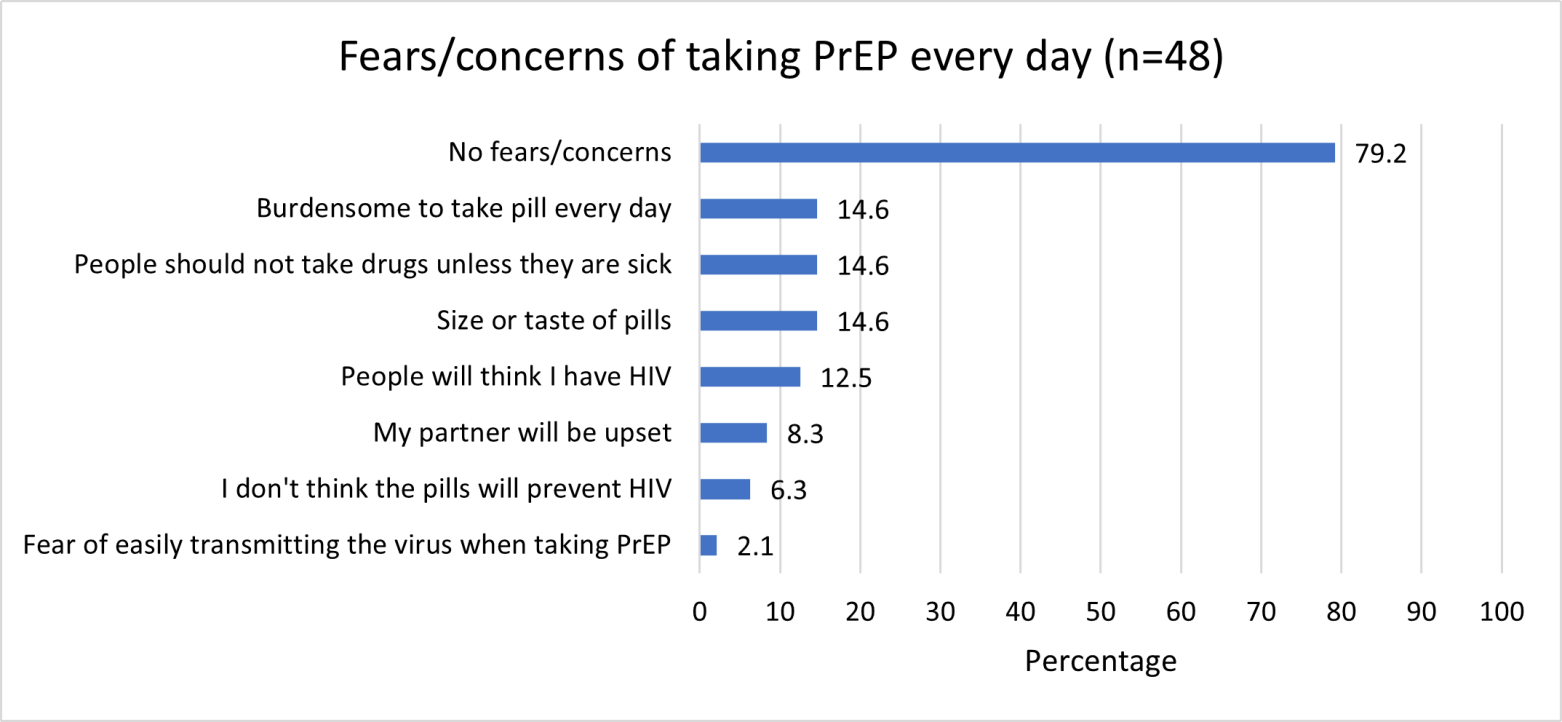
AGYW fears and concerns of taking PrEP every day (N = 48).

Only 26.0% of participants (N = 52) had ever heard of PrEP, mostly from community activists (14/52, 26.9%). In the logistic regression model, no variables were significant with our population. Only 31.3% were interested in taking PrEP; 20.8% said they were unsure and 47.9% of participants said they were very uninterested. Participants were asked to choose one response from the following that best matched their understanding of PrEP: taking medicine after you are exposed to HIV to prevent getting HIV (34.6%), daily medicine you take when you are HIV negative to prevent you from getting HIV regardless of if you have been exposed to it (65.4%), never heard of PrEP (9.6%), or other (all incorrect understandings, 15.4%). Those who had heard of PrEP had an accurate understanding of why PrEP is used (65.4%). Most (79.2%) participants who had heard of PrEP prior to the survey but had never used it had no major concerns around taking PrEP daily. The reasons that participants gave for not wanting to take PrEP in general included the size or taste of the pills, not wanting to take drugs when they are not sick, and the burden of taking a daily pill (Fig 2). More than a third (37.5%) were worried about side effects of taking PrEP and 41.7% were unsure about its effectiveness in preventing HIV.

Four participants (2.0%) had ever used or were currently using PrEP, and only eight AGYW (4.0%) reported knowing someone who takes PrEP. Among the four participants who had ever taken PrEP, three of them reported that they started taking PrEP because they were offered PrEP by a healthcare provider. Only one participant was still taking PrEP at the time of the survey; the reasons given by the others who had discontinued were just wanting to stop, not wanting to engage in follow-up, or because they thought they were no longer at risk of HIV. The participant still taking PrEP reported the reason for continuing PrEP was the empowerment they felt while taking it.

**Fig 3:**
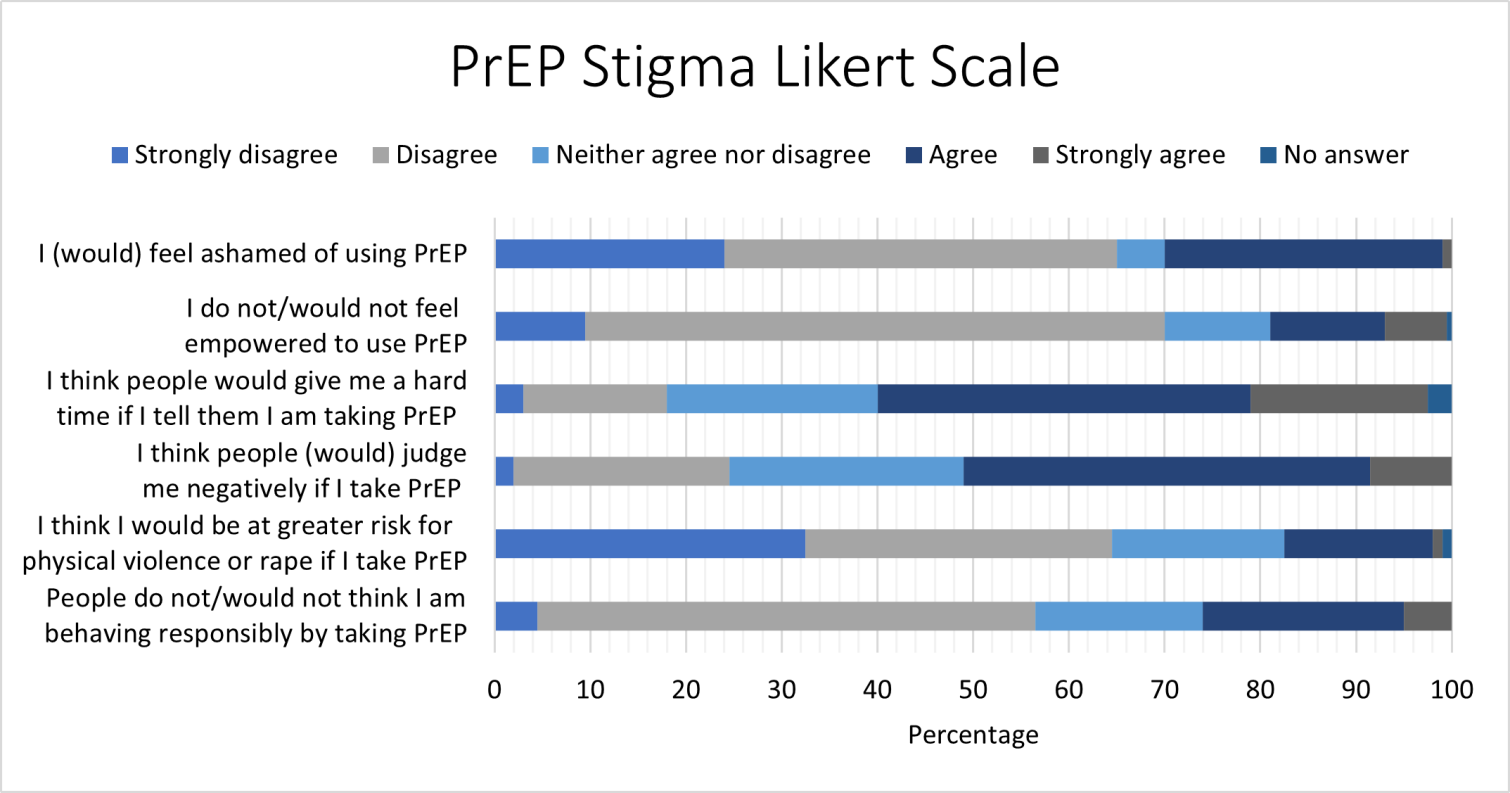
Likert scale of statements related to stigma of PrEP use.

The survey also included six statements regarding PrEP stigma (Fig 3) for all participants to reflect on, responding with “strongly disagree,” “disagree,” “neither agree nor disagree,” “agree,” “strongly agree,” or “no answer.” The statements with the highest scores of stigma were “I think people would give me a hard time (such as make fun of me or talk badly about me) if I tell them I am taking PrEP” (57.5% agreed) and “I think people (would) judge me negatively if I take PrEP” (51.0% agreed). On the other hand, 70.0% of participants agreed with the statement “I (would) feel empowered to use PrEP.”

### PrEP availability

When asked about interest in different PrEP modalities, 63.5% of all participants showed interest in using daily oral PrEP, 56.0% were interested in on-demand oral PrEP, 75.5% were interested in long-acting injectable PrEP (IM-LA), and only 30.0% were interested in a PrEP vaginal ring. Participants considered IM-LA PrEP (63.5%) and daily oral PrEP (22.0%) as the most appropriate methods of PrEP for young people (aged 15–25 years).

The survey also asked about the participants’ preferences of the logistics of taking daily oral PrEP. The majority of participants (60.0%) stated that ideal intervals of picking up PrEP would be every three months or every six months. Most respondents (56.0%) did not want PrEP to be a standalone service, and 70.0% preferred that PrEP be integrated into another service. The preferred places to pick-up medication refills were at clinic check-ups and at the health facility (67.0%). Participants interested in PrEP support group activities (N = 150) expressed desire activities to be at their local health facility (48.0%) and consist of medication refill pickups (81.3%), HIV testing and counseling (70.0%), and adherence counseling (73.3%), and sexual and reproductive health counseling (90.7%). Most participants showed preference for support groups with both males and females around their age. However, younger AGYW were more likely to want only women in their support groups (p = 0.004, RR = 2.2, 95% CI: 1.2-3.8).

## Discussion

Our survey describes the demographic characteristics of AGYW 15–25 years of age engaged in care at Adolescent and Youth Friendly Service (SAAJ) clinics in Nampula, Mozambique as well as their knowledge, attitudes, and practices related to family planning and HIV prevention methods, specifically PrEP. Our findings highlight the complex challenges faced by this population due to societal norms including social inequalities, high-risk sexual behavior, and low engagement with and adherence to contraception and HIV prevention methods. The setting of our survey, Nampula, Mozambique, has been previously characterized as an economically underprivileged city in one of the world’s poorest countries with high rates of sexual activity among AYA, which is reflected in our survey [31,32]. Overall, survey participants had low levels of education and employment. Participants had high cell phone ownership but limited access to household facilities.

Most survey participants reported being sexually active with a median age of 17 years at first sex and a median age of 18 years at first pregnancy. Of those who reported sexual activity, 81.0% said that they had sex because they wanted to try it, but a concerning 17.5% reported that the first time they had sex was forced – two-thirds (9.0% of all participants) of these were pressured into sex and one-third (4.5% of all participants) were physically forced. These results mirror other studies of adolescents in SSA; examples being one study among AGYW in South Africa reported that 70.7% wanted their first sexual experience, while 16.7% reported not wanting it and 3.2% were forced/raped [33] and another in Uganda reported that 85.7% were willing to have sex for the first time while 13.1% were not willing at all [34]. Despite the majority of adolescents reporting willingness to have sex for the first time, support is needed for those reporting being forced or coerced at first sex. Though programs aiming to reduce gender-based violence and HIV acquisition have been implemented in SSA and have been successful [35,36], this does not mitigate the need for consistent support for survivors who have already experienced sexual violence.

Our survey reported that less than 2.0% of AGYW had become pregnant before the age of 15. A 2015 survey reported decreasing rates of AGYW giving birth before the age of 15 in Eastern Africa, with a rate of 2.5% [37]. Despite this encouraging statistic as well as the fact that survey respondents were familiar with a variety of family planning methods, the number of surveyed AGYW who reported use of modern contraception was low. Less than half of those surveyed reported currently doing something to delay or avoid getting pregnant, with 30.5% reporting that they were not using anything. Furthermore, those surveyed reported infrequent condom usage and withdrawal as a commonplace family planning method, keeping these AGYW at high risk of HIV and unplanned pregnancies [38]. Reasons for not using any method included not having regular intercourse, wanting to become pregnant, and being afraid of contraceptives and their side effects. These results are supported by other studies exploring why women do not use contraception [39–41]. Unsurprisingly, the older age group were more likely to have heard of and used different family planning methods. Other studies have also reported that older AGYW tended to be more familiar with modern contraceptive methods [42]. Even though our population has shown high knowledge of both traditional and modern contraceptive methods, the low and inconsistent use of these methods is a concern that needs to be addressed by existing public sector family planning programs including those catering to AGYW in the SAAJ clinics. Understanding the reasons why AGYW are not using contraception, including addressing associated fears, is critical to better tailor family planning programs to the unique needs of AGYW and encourage uptake and adherence [41,43–46]. Additionally, though it was not expressed by survey participants, it is also important to note that healthcare workers themselves may be discouraging AGYW from seeking contraceptive services by exhibiting unfriendly and judgmental attitudes or not ensuring confidentiality between the patient and provider [43,45].

National rollout of PrEP in Mozambique started in 2021, targeting AGYW in particular. However, although survey participants were recruited from clinics currently providing PrEP and PrEP education, only 26.0% of participants had ever heard of PrEP a year after provision of PrEP began. A number of our survey respondents heard about PrEP through community activists. Another study done in Kenya showed that widespread community campaigns played a role in increasing PrEP awareness, though the information shared was often seen as vague and confusing and participants sought out more information at clinic visits [47]. This highlights the importance of including detailed information in these campaigns, as well as personal interaction, such as with community activists, where individual questions can be answered quickly and accurately. In our survey, younger and older participants were equally likely to have heard of PrEP. This is consistent with other studies that did not find an association between age and PrEP knowledge [20,22]. Only four survey participants (2.0% of all participants), all 19 years or older, had ever used PrEP. Of this small group, only one was still using PrEP, which, despite the extremely low sample size, reflects a 25.0% continuance rate. The participants themselves showed an overall interest in using PrEP and reported lower feelings of internalized stigma when considering their opinion of themselves potentially using PrEP but reported higher levels of externalized stigma when considering the potential of others judging them, including parents and peers, who knew they used PrEP. Qualitative studies done in both Zimbabwe and South Africa suggest that parents may be reluctant to allow their child to initiate PrEP due to fear of their child being labeled as promiscuous or presumed to be HIV-infected [48,49]. The AGYW participants included in the Zimbabwe study reported difficulties in asking or notifying their parents about PrEP because of the desire to be seen as a good child and to not be seen as sexually promiscuous, despite their personal desire to take PrEP [50]. Other research has shown that community stigma negatively affects adherence to PrEP due to misunderstanding and lack of education around what PrEP actually is [48]. An additional source of perceived judgement are the clinic staff, who may come off as harsh, judgmental, or untrustworthy [50,51]. It is critical that PrEP messaging is created so that the language is non-stigmatizing, especially for those who have not heard of PrEP before to avoid this barrier of stigma to PrEP uptake [52,53].

Remarkably, more than half of participants in our survey agreed that people would think that they are behaving responsibly by taking PrEP. Previous research suggests that this effect is particularly pronounced when receivers of care and people around them are appropriately informed; Duby et al shares stories of parents changing their minds about their children taking PrEP after being meaningfully engaged and properly educated [48].

Despite fearing judgement from others, the study population reflected high levels of interest in using PrEP, especially IM-LA (75.5%) and daily oral PrEP (63.5%). Interestingly, the most acceptable methods of PrEP (IM-LA, daily oral pill) were similar to the more acceptable methods of contraception, which were injectables and the pill. Other studies have also reported that for both contraceptive and PrEP methods, injectables are more popular than daily pills, supporting our results [48,54,55]. Our study population showed markedly less interest in the PrEP vaginal ring. The IUD was also reported as being unacceptable and feared in the study population, with participants reporting fears of the IUD getting lost in the uterus/body, insertion causing damage to the vagina, and the IUD causing infertility. Due to the similarities in placement and insertion method of the IUD and vaginal ring, we can infer that the concerns surrounding use of the vaginal ring mirror those of the IUD. For example, in a study in Kenya and South Africa that looked at HIV prevention product ratings before and after three months of use, participants who were assigned the vaginal ring expressed concerns about the large size of the ring and the discomfort and difficulty of inserting the ring [56]. These results agree with a study focused on key populations in South Africa, where IM-LA was much more preferred over the Dapivirine vaginal ring [57]. Another study in South Africa that gave all participants an opportunity to try the vaginal ring and one other contraceptive method (injectable or daily pill) also showed strong support for injectables (54.5%) [58]. Interestingly, after trying both methods, the vaginal ring (39.4%) was preferred over taking daily pills (6.1%), which shows that experience and education is important in helping women decide which method works best for them. In the Kenya and South Africa study, participants who received injections or the vaginal ring rated their products higher after use than those who took pills [56]. Studies have shown that unwillingness and fear of trying methods such as the IUD or vaginal ring is due to inadequate knowledge, lack of trust in preventive methods, and fear of side effects [41,59,60]. Participants in the Kenya and South Africa study who originally expressed concerns regarding the size and insertion of the vaginal ring reported that their experience with the vaginal ring assuaged their fears [56]. Adapting lessons learned from expanding provision of oral PrEP and contraception, including broadening engagement of peers and community members, making information more accessible, and providing consistent support, will help to placate fears of potential users as well as manage spread of misinformation among communities [41,59,60].

A large part (82.5%) of our population preferred that PrEP refills and support group activities occur at the clinic or local health facility. A qualitative study done in South Africa with potential PrEP users showed similar results, where participants chose local healthcare facilities and pharmacies to be acceptable places for service delivery points [57]. The majority (70.0%) of participants also expressed a preference for PrEP services to be integrated into other health services. Analysis of oral PrEP introduction and subsequent expansion in sub-Saharan African countries shows that provision of PrEP at youth-friendly settings as well as integration of PrEP services into other health services have been successful in achieving high PrEP initiation rates [61]. Other studies have found success in providing PrEP by integrating PrEP distribution with services such as sexually transmitted infection testing, contraception distribution, and maternal and child health and family planning services [62–64]. Responses from our survey reflected strong preference for peer support groups. Social support from peers is a strong motivator for contraceptive use among young people [60] and have been shown to be successful among AGYW [61].

### Study strengths and limitations

As far as we know, our study is the first survey to look at the knowledge, attitudes, and practices regarding family planning and PrEP among AGYW in urban areas of Mozambique. As this population may be at particular risk for HIV infection, it is important to explore and understand the barriers and facilitators that may exist related to accessibility of sexual and reproductive health services and HIV prevention resources among this population. As new PrEP modalities, including long-acting PrEP agents, are introduced in this setting, data from this survey may be useful to inform future PrEP programming and improving current PrEP delivery systems for this group. A limitation of this study is that participants were recruited from clinics that provide sexual and reproductive health services to AGYW, as such they may be more informed about contraception and HIV prevention. This also may have led to social desirability bias as study recruitment and survey administration took place at the SAAJ clinics, and participants may have felt pressure to answer a certain way lest their responses affect their healthcare. Another limitation is the small sample size; 66% of Mozambique’s population, or approximately 22.6 million people, is under 25 years of age, and only 200 AGYW were surveyed [65]. Although the survey instrument included some open-ended questions, most of the questions were strictly quantitative. Future research could include focus group discussions or in-depth interviews to further explore these topics and collect more detailed information about AGYW attitudes and practices. Future studies should target the general AGYW population, including those who are not attending SAAJ clinics, key populations, and other marginalized people. It will also be important to engage AGYW living with HIV and explore the HIV continuum of care, as well as their knowledge, attitudes, and practices toward HIV transmission prevention practices.

## Conclusion

The results from this study reveal the continued need for widespread education and improved accessibility of both contraception and PrEP among AGYW in Nampula, Mozambique. Low rates of contraception usage and low PrEP awareness imposes a burden on sexually active AGYW and leave them vulnerable to poor sexual health outcomes and HIV acquisition. Adolescent-friendly clinics need to address AGYW concerns around different modalities of contraception in addition to PrEP usage to increase both engagement and adherence to these prevention methods.

## Supporting information

S1 Table Sources of survey sections. (DOCX)

S1 Checklist Inclusivity in global research. (DOCX)
