# Supplementary material for: Contraception and PrEP knowledge, attitudes, and practices among adolescent girls and young women in Nampula, Mozambique
Source: PLOS Glob Public Health. 2025 Jun 16;5(6):e0004746. doi: 10.1371/journal.pgph.0004746 (PMC12169551; doi:10.1371/journal.pgph.0004746)
Supplement: S1 Table — (DOCX) [file pgph.0004746.s001.docx]

**S1 Table. Sources of survey sections**

| **Survey Section** | **Source** |
| --- | --- |
| Demographics | DHS Section 1 (22) |
| Education | DHS Section 1 (22) |
| Employment | DHS Section 1 (22), CombinADO survey (21) |
| Household Characteristics | CombinADO survey (21) |
| Sexual Behavior | GSHS Core-Expanded Questions for the Module on Sexual Behaviours that Contribute  to HIV Infection, Other STI, and Unintended Pregnancy (24)  Cleland Sections 3 7, 9 (23) |
| Contraception Knowledge, Attitudes, and Practices | DHS Section 3, 7 (22) |
| Relationships and Pregnancy | DHS Section 2, 4 (22) |
| HIV Testing | DHS Section 10 (22) |
| HIV Knowledge | DHS Section 10 (22) |
| PrEP Knowledge and Attitudes | Sila “PrEP Initiation and Continuation”, “PrEP Adherence” (25) |
| PrEP Stigma | Sila “PrEP Attitudes and Acceptability” (25) |
| PrEP Modality Preference | DHS Section 3, 7 (22) |
| Sexual and Reproductive Services and COVID-19 | Kenya KAP survey |
